# Hsp70 Suppresses Mitochondrial Reactive Oxygen Species and Preserves Pulmonary Microvascular Barrier Integrity Following Exposure to Bacterial Toxins

**DOI:** 10.3389/fimmu.2018.01309

**Published:** 2018-06-12

**Authors:** Xueyi Li, Yanfang Yu, Boris Gorshkov, Stephen Haigh, Zsuzsanna Bordan, Daniel Weintraub, Radu Daniel Rudic, Trinad Chakraborty, Scott A. Barman, Alexander D. Verin, Yunchao Su, Rudolf Lucas, David W. Stepp, Feng Chen, David J. R. Fulton

**Affiliations:** ^1^Vascular Biology Center, Medical College of Georgia at Augusta University, Augusta, Georgia; ^2^Department of Forensic Medicine, Nanjing Medical University, Nanjing, Jiangsu, China; ^3^Department of Pharmacology and Toxicology, Medical College of Georgia at Augusta University, Augusta, GA, United States; ^4^Institute for Medical Microbiology, Justus-Liebig University Giessen, Giessen, Germany

**Keywords:** pneumolysin, endothelial barrier, reactive oxygen species, mitochondria, Hsp70

## Abstract

Pneumonia is a leading cause of death in children and the elderly worldwide, accounting for 15% of all deaths of children under 5 years old. *Streptococcus pneumoniae* is a common and aggressive cause of pneumonia and can also contribute to meningitis and sepsis. Despite the widespread use of antibiotics, mortality rates for pneumonia remain unacceptably high in part due to the release of bacterial toxins. Pneumolysin (PLY) is a cholesterol-dependent toxin that is produced by *Streptococcus*, and it is both necessary and sufficient for the development of the extensive pulmonary permeability edema that underlies acute lung injury. The mechanisms by which PLY disrupts the pulmonary endothelial barrier are not fully understood. Previously, we found that reactive oxygen species (ROS) contribute to the barrier destructive effects of PLY and identified an unexpected but potent role of Hsp70 in suppressing ROS production. The ability of Hsp70 to influence PLY-induced barrier dysfunction is not yet described, and the goal of the current study was to identify whether Hsp70 upregulation is an effective strategy to protect the lung microvascular endothelial barrier from G^+^ bacterial toxins. Overexpression of Hsp70 *via* adenovirus-mediated gene transfer attenuated PLY-induced increases in permeability in human lung microvascular endothelial cells (HLMVEC) with no evidence of cytotoxicity. To adopt a more translational approach, we employed a pharmacological approach using geranylgeranylacetone (GGA) to acutely upregulate endogenous Hsp70 expression. Following acute treatment (6 h) with GGA, HLMVECs exposed to PLY displayed improved cell viability and enhanced endothelial barrier function as measured by both Electric Cell-substrate Impedance Sensing (ECIS) and transwell permeability assays compared to control treated cells. PLY promoted increased mitochondrial ROS, decreased mitochondrial oxygen consumption, and increased caspase 3 cleavage and cell death, which were collectively improved in cells pretreated with GGA. In mice, IP pretreatment with GGA 24 h prior to IT administration of PLY resulted in significantly less Evans Blue Dye extravasation compared to vehicle, indicating preserved endothelial barrier integrity and suggesting that the acute upregulation of Hsp70 may be an effective therapeutic approach in the treatment of lung injury associated with pneumonia.

## Introduction

Pneumonia is a pulmonary infection that can affect people of all ages, but is most severe in the elderly, children, and the immunocompromised. Infection and subsequent inflammation compromise the function of lung endothelial and epithelial barriers resulting in alveolar flooding, impaired gas exchange, and eventually lung consolidation, which collectively underlie the development of acute lung injury (ALI) and its more severe form, acute respiratory distress syndrome (ARDS) (1). Multiple pathogens can promote pneumonia, including bacteria, fungi, and viruses. Bacteria, in particular Gram positive (G^+^) bacteria, are the most common cause (2, 3). Currently there are no effective pharmacological approaches for ALI/ARDS and patients treated with anti-inflammatory steroids treatment remain at significant risk of mortality (4).

A hallmark feature of ALI/ARDS is dysfunction of the pulmonary microvascular endothelial barrier resulting in an imbalance of Starling’s forces and the passage of excess fluid into the alveoli (1). The mechanisms underlying the loss of the microvascular barrier function are complicated and involve diverse mechanisms in multiple cell types which can vary depending on the causative agent. A major cause of ALI is G^+^ bacteria including *Streptococcus pneumoniae* which accounts for up to half of all community-acquired pneumonia (CAP) cases in the US and CAP is the most frequent cause of ARDS (3). Greater than 500,000 yearly cases of pneumonia and 25,000 pneumococcal-related deaths are reported in the US alone, resulting in a health-care burden that exceeds $5 billion dollars (5). The first-line treatment for *Streptococcus* pneumonia is antibiotic therapy. However, the onset of ARDS is resistant to antibiotics and paradoxically, bacteriolytic antibiotics can exacerbate lung injury (6). One likely reason for this is the G^+^ toxin, pneumolysin (PLY), which is produced in *Streptococcus* and released by autolysis and in greater amounts in the presence of antibiotics that compromise the bacterial cell wall (7). PLY is a 53-kDa intracellular protein which belongs to the cholesterol-dependent cytolysin family (8). Upon binding to cholesterol molecules on the cell membrane of target cells, PLY induces the macromolecular assembly of ring shaped pores that promote calcium influx and alter intracellular signaling (9, 10). Subsequent to these changes, G^+^-toxins robustly increase the intracellular production of reactive oxygen species (ROS) (6, 7, 11–13). Elevated ROS have been shown to have important roles in regulating a number of physiological and pathophysiological events, including cell apoptosis, survival, proliferation and migration, cell metabolism, DNA damage, inflammation, and disruption of the endothelial barrier (14). The major sources of ROS in endothelial cells are the NADPH oxidases (NOX enzymes), uncoupled eNOS, and the mitochondria. G^+^-toxins have been reported to activate PKC and alter eNOS fidelity to disrupt the balance of nitric oxide and superoxide (13), activate NADPH oxidase (7), and increase mitochondria-derived ROS (mtROS) (15). There are also significant interactions between these ROS generating systems, where mitochondrial ROS can activate NOX enzymes and *vice versa* and increased ROS can lead to eNOS uncoupling and ROS production. Mitochondrial DNA (mtDNA) is highly sensitive to ROS and loss of mtDNA integrity can result in mitochondrial dysfunction, ATP deprivation, and cell apoptosis (16–19). NADPH-derived ROS have been shown to promote oxidative damage of mitochondrial proteins in particularly, complex I and complex II, which results in increased mitoROS production (20). Increased mitoROS can activate NADPH oxidase promoting a feed-forward relationship (20, 21). NOX2 has been identified as a potential target for mitochondrial superoxide production in endothelial cells (21, 22) and increased mtROS, secondary to a partial deficiency of mitochondrial superoxide dismutase, can trigger a cytosolic oxidative burst (21). On the other hand, inhibitors that reduce mtROS can also attenuate cytosolic ROS (21–23). ROS can deplete BH4 and alter the S-glutathionylation of eNOS, compromising NO formation and increasing ROS production (24, 25).

Mitochondria are increasingly recognized for their contributions to inflammation, and mtROS is a key factor mediating this process in endothelial cells in response to both physiological and pathophysiological stressors (16, 19). Along with the activation of NOX enzymes, mtROS also promote the activation of endothelial cells and increase proinflammatory cytokines (26) in a manner synergistic with cytosolic ROS. Antioxidants targeted to the mitochondria can reduce endothelial inflammation in hypertension animal models (27). The inflammatory process during pneumonia is complex, and different in the young versus elderly patients. In aged patients, inflammation may initiate at slowly in the early phase compared to young patients, but is more robust and enduring in the later stages (28). This deregulated inflammatory response is a risk factor for death in elderly patients, and broad anti-inflammatory strategies can result in compromised elimination of pathogens.

Heat-shock proteins (Hsp) are intracellular “chaperones” that guide the behavior (folding, function, and fate) of newly synthesized proteins and also associate stably with many signaling molecules (29). They are critical to the maintenance of cellular homeostasis under both physiological and stressed conditions (30). The major chaperones are Hsp90 and Hsp70 which together with co-chaperones mediate protein folding, complex assembly, intracellular transport, and also degradation (31, 32). Previously, we and others have shown that Hsp90 binds specifically to Nox proteins to regulate enzyme stability and ROS production (33, 34). We also found that Hsp90 inhibitors can robustly upregulate Hsp70 expression and that Hsp70 alone can potently suppress NOX-derived ROS production in human pulmonary arterial endothelial cells (35).

Hsp70 has also been shown to be important for cell survival (36) and regulating mitoROS and mtDNA integrity (37), but whether it can provide protection from PLY-induced endothelial injury and loss of endothelial barrier function by suppressing ROS is not yet known. Therefore, the goals of this study were to assess the importance of Hsp70 in protecting the pulmonary microvascular endothelium from PLY, to identify the underlying mechanisms, and to advance the possibility of targeting Hsp70 as a promising therapy for *Streptococcus*-induced pneumonia.

## Materials and Methods

### Cells and Reagents

Human lung microvascular endothelial cells (HLMVECs) were obtained from Lonza. Cells were cultured with 5% CO_2_ at 37°C using EBM-2 MV medium supplemented with EGM-2 MV that was purchased from Lonza. All of the *in vitro* experiments were performed using passage 3 to passage 5 HLMVECs. PLY was a gift from Dr. Trinad Chakraborty (Institute for Medical Microbiology, Justus-Liebig University, Giessen, Germany) PLY was purified from a recombinant *Listeria innocua* 6a strain expressing LPS-free PLY. Geranylgeranylacetone (GGA, Sigma) was prepared in DMSO. Tempol, LPS, Glucose, pyruvate, and l-glutamine were obtained from Millipore Sigma. Oligomycin, FCCP, and antimycin were provided in the Seahorse XF Cell Mito Stress Test Kit from Agilent. FITC dextran (Fluorescein isothiocyanate–dextran 4000 and Fluorescein isothiocyanate–dextran 70000) were obtained from Sigma. Antibodies for western blotting included Hsp70 from BD Bioscience, cleaved caspase 3, NF-κB, and GAPDH were from Cell Signaling and Hsp90 from BD Bioscience. Nox_1_ antibody from Sigma. GFP and Hsp70 adenoviruses were generated in house using established methodologies (38, 39).

### Animals

8- to 10-weeks-old male C57BL6 mice, weighing 19–21 g were obtained from Harlan and were kept at the animal facilities at Augusta University. All animal studies conformed to National Institutes of Health guidelines. The experimental procedure was approved by the Augusta University Institutional Animal Care and Use Committee.

### Assessment of Pulmonary Vascular Barrier Function *In Vivo*

Mice were pretreated with 500 mg/kg GGA (in ethanol) administered IP 24 h prior to toxin instillation. Mice were anesthetized with IP ketamine (150 mg/kg) and acetylpromazine (15 mg/kg), the trachea was exposed and PLY (60 ng) instilled IT for 6 h *via* a 20-gauge catheter. Evans blue dye (EBD)/albumin mixture (30 mg/kg in saline; 0.5% EBD conjugated to 4% BSA, Fraction V; Sigma-Aldrich, St. Louis, MO, USA) was injected into the tail vein, 2 h prior the conclusion of the experiment, in order to assess vascular leak. The lungs were homogenized, incubated with formamide (18 h at + 60°C), and centrifuged at 5,000 × *g* for 30 min. The optical density of the supernatant was determined spectrophotometrically at 620–750 nm. The concentration of extravasated EBD in the lungs was calculated by using a standard curve (micrograms of EBD per gram of wet lung tissue), as described previously (40).

### Western Blotting

Cells were washed three times with HBSS before lysing with Laemmli sample buffer. Lysed cells were briefly sonicated to ensure protein extraction, proteins size fractionated by SDS PAGE and transferred to nitrocellulose membranes. Membranes were incubated overnight at 4°C with antibodies diluted to the manufacturer’s specifications. Secondary IgG antibodies (Invitrogen) conjugated with horseradish peroxidase were used to detect antigen–antibody complexes.

### Cell Viability Assay

Cell viability was assessed using the Muse Cell Analyzer (Millipore Sigma). HLMVECs were seeded at a density of 6 × 10^5^ in 6-well plates with complete EBM-2 MV culture medium with or without 30 µM GGA or DMSO for 6 h. PLY was added to dishes 4 h before the viability test. The percentage of live HLMVECs was determined using the Muse cell count and viability kit (Millipore Sigma). In brief, 50 µl of suspended HLMVECs was mixed with 450 µl of count and viability reagent, gently mixed, and injected into the Muse Cell Analyzer. Statistical analysis was performed based on three independent experiments. *p* Value was compared to the cell treated with GGA plus PLY group.

### Endothelial Cell Permeability Assays

#### Transwell Cell Permeability Assay

Sub confluent HLMVECs were split into 24 transwell inserts with a 0.4-µm pore sized filter. Following seeding, cells were maintained in complete EBM-2 MV medium and then treated for overnight with or without 30 µM GGA. Prior to exposure to PLY, complete medium was removed, and HLMVECs were washed carefully with HBSS three times before transitioning to FITC-dextran containing serum-free medium. Fresh FITC-conjugated dextran was prepared using serum-free medium and 50 ng/ml PLY was added to the endothelial cells. The lower compartment was filled with 1.5 ml of serum-free medium without FITC-dextran. At each time point, 50 µl of lower chamber medium was transferred to a 96-well plate for quantitation. The amount of FITC-dextran permeating through the HLMVEC monolayer into the lower chamber was measured using plate reader with excitation wave length 488 nm and emission wave length 520 nm. Three independent experiments were performed and the data are shown as mean ± SEM.

#### Transendothelial Electrical Resistance Measurements

Transendothelial electrical resistance was measured in HLMVEC using electric cell-substrate impedance sensing equipment (ECIS). HLMVECs were split into ECIS array chambers (8W10E) at a density of 1,000 cells per well according to the manufacturer’s instructions. On the following day, complete medium was removed, and cells were washed with HBSS for three times. The cells were then cultured in serum-free medium and exposed to PLY to initiate changes in barrier function. Normalized resistance (Ohms) representing HLMVEC barrier integrity was recorded for up to 3 h. Data were aggregated as the mean normalized resistance of eight individual wells.

### MitoSOX Assay

Human lung microvascular endothelial cells were transferred into glass-bottomed dishes and cultured in complete EBM-2 MV medium and pretreated with or without 30 µM GGA. On the following day, cells were washed in HBSS, the culture medium changed to serum-free EBM-2. MitoSOX Red and MitoTracker Green were diluted with pre-warmed appropriate medium to a final concentration of 5 µM and 250 nM, respectively. Medium containing MitoSOX Red and MitoTracker Green was applied to the HLMVECs, stimulated with PLY and cells were incubated in 37°C for 15 min prior to observation using a Zeiss 780 inverted confocal microscope. MitoSOX Red mitochondrial superoxide indicator signal was measured at excitation/emission wavelength of 510/580 nm, and MitoTracker Green was detected using an excitation/emission wavelength of 488/516 nm. Wells without dyes were tested for MitoSOX Red and MitoTracker Green signals, respectively, as background signals. Nuclear blue was used to stain the nucleus. Equivalent experiments were performed using a fluorescent plate reader (POLARstar OMEGA) for quantification. After subtracting the background from the signal recorded in the presence of each dye, the ratio of MitoSOX Red signal over MitoTracker Green was used to determine the amount of mitochondrial ROS. The data are presented as fold change over control HLMVEC cells.

### Mitochondrial Stress Assay

Mitochondrial stress in HLMVEC was assessed using the Seahorse XF96 analyzer. Low passage number HLMVECs were cultured in the XF96 well plate at a density of 7.0 × 10^3^ cell per well in complete EBM-2 MV medium with or without GGA (30 µM) overnight. The sensor cartridge of a XF96 seahorse plate was hydrated overnight at 37°C in a non CO_2_ incubator. The following day, fresh seahorse assay medium was prepared with the addition of glucose (10 mM), pyruvate (1.0 mM), and l-glutamine (2 mM). The pH of the medium was adjusted to 7.4 using NaOH. Cell confluency was assessed under light microscopy, and the plate was incubated in a non CO_2_ incubator for 1 h. After calibration of the sensor cartridge, the XF96 plate was placed into the seahorse instrument and OCR measured as pmole per minute per cell. Analysis of mitochondrial function was made using changes in OCR in the presence of oligomycin (inhibits mitochondrial ATP synthase), and phenylhydrazone (FCCP, mitochondrial membrane potential) which were injected at final concentrations of 1.0 µM each. Lastly, antimycin A was injected at a final concentration of 0.5 µM to inhibit both complex I and III. After each injection, the Seahorse instrument measured OCR for three times at five time points. The average of three measurements was used for data analysis.

### Statistical Analysis

Data are presented as means ± SEM. Single comparisons were made using a Student’s *t*-test and multiple comparisons were made using one way ANOVA with an appropriate *post hoc* test (Tukey). *p* < 0.05 was considered a statistically significant difference.

## Results

### Hsp70 Protects HLMVECs From PLY-Induced Barrier Destruction

**Figure 1:**
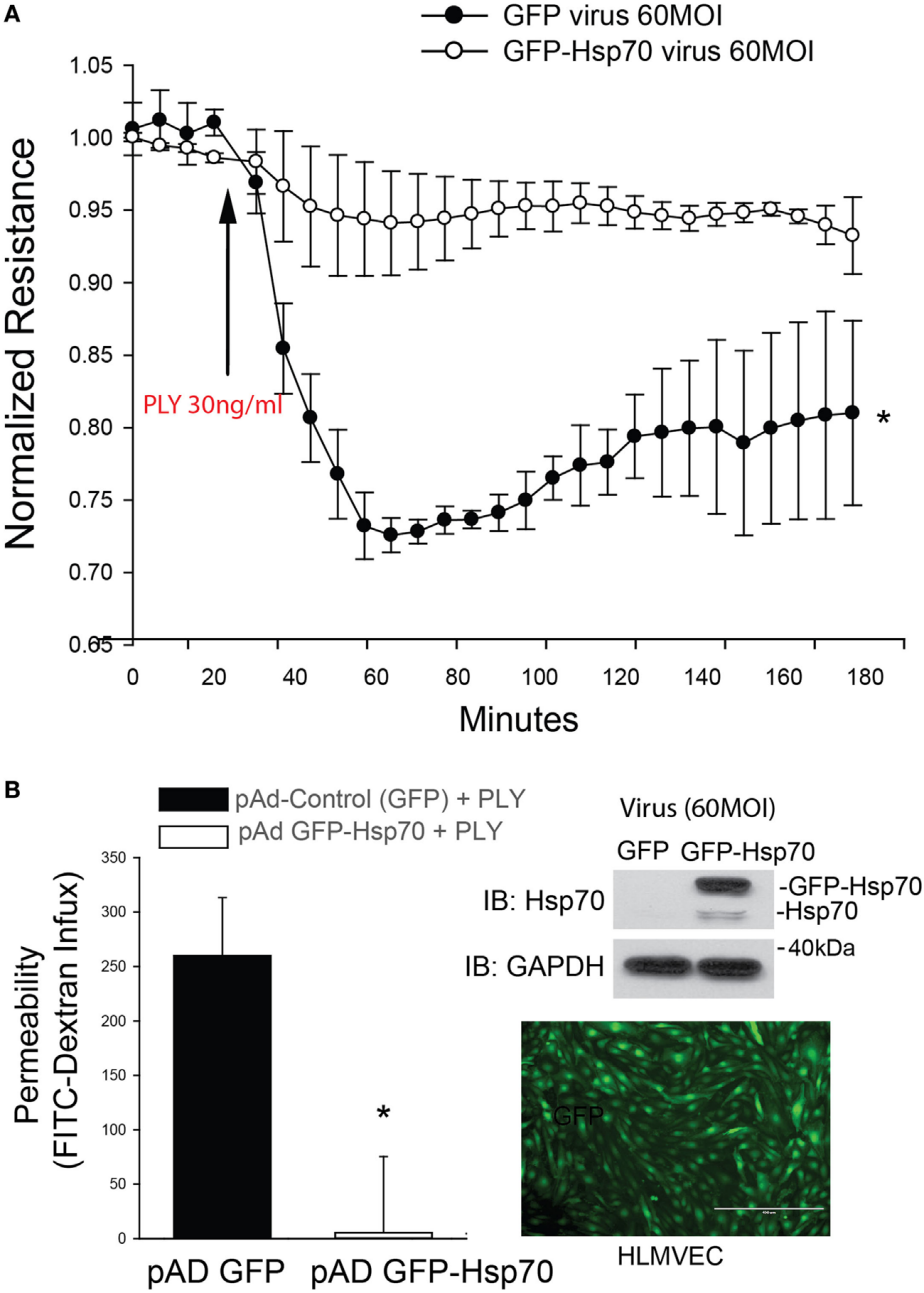
Hsp70 provides robust protection against pneumolysin (PLY)-induced EC barrier disruption. In panel **(A)** human lung microvascular endothelial cells were transduced with pAD-GFP (Control) or pAD-GFP-Hsp70 at 60MOI and 48 h later, cells were assessed for barrier function using transendothelial resistance as monitored by electric cell-substrate impedance sensing (ECIS) in the presence and absence of PLY (30 ng/ml). In panel **(B)** (left) HLMEC were grown in transwells and similarly transduced with GFP or GFP-Hsp70 and the flux of FITC-dextran (70 kDa) into the bottom chamber determined using a fluorescent plate reader. On the right (top panel) relative expression of the GFP-Hsp70 transgene relative to endogenous and (bottom panel) image showing the expression pattern of GFP in transduced cells. Data are shown as mean ± SEM (*n* = 3 wells for each treatment). *p < 0.05 versus control.

Pneumolysin has been well documented to disrupt barrier function in HLMVEC and increase endothelial permeability (12, 13, 41, 42). To determine the effect of Hsp70 on barrier function, HLMVECs were transduced with adenoviruses encoding Hsp70 (pAd Hsp70-GFP, 60MOI) or GFP (Control, 60MOI) overnight and then transferred to an ECIS plate. HLMVECs transduced with the Hsp70 adenovirus were significantly protected from PLY-induced barrier dysfunction as compared to control cells (Figure 1A). The protective effect of Hsp70 on barrier function was then confirmed using a distinct approach, the transwell permeability assay. HLMVECs transduced with pAd Hsp70-GFP or pAd-GFP were seeded into the upper compartments of transwell plates. FITC-dextran (70 kDa) was added to the medium in the upper compartments of test wells, the background wells remained without dextran and all were treated with PLY. FITC-dextran flow through was measured in the lower compartments using a fluorescence capable plate reader (BMG POLARstar Omega). Consistent with the ECIS assay, HLMVEC expressing Hsp70 were protected from PLY-induced barrier destruction (Figure 1B, left panel). Expression of transgenes was confirmed using fluorescent microscopy and western blot (Figure 1B, right panel).

### The Pharmacological Inducer of Hsp70 Expression, GGA Protects HLMVECs From PLY-Induced Increases in Permeability

**Figure 2:**
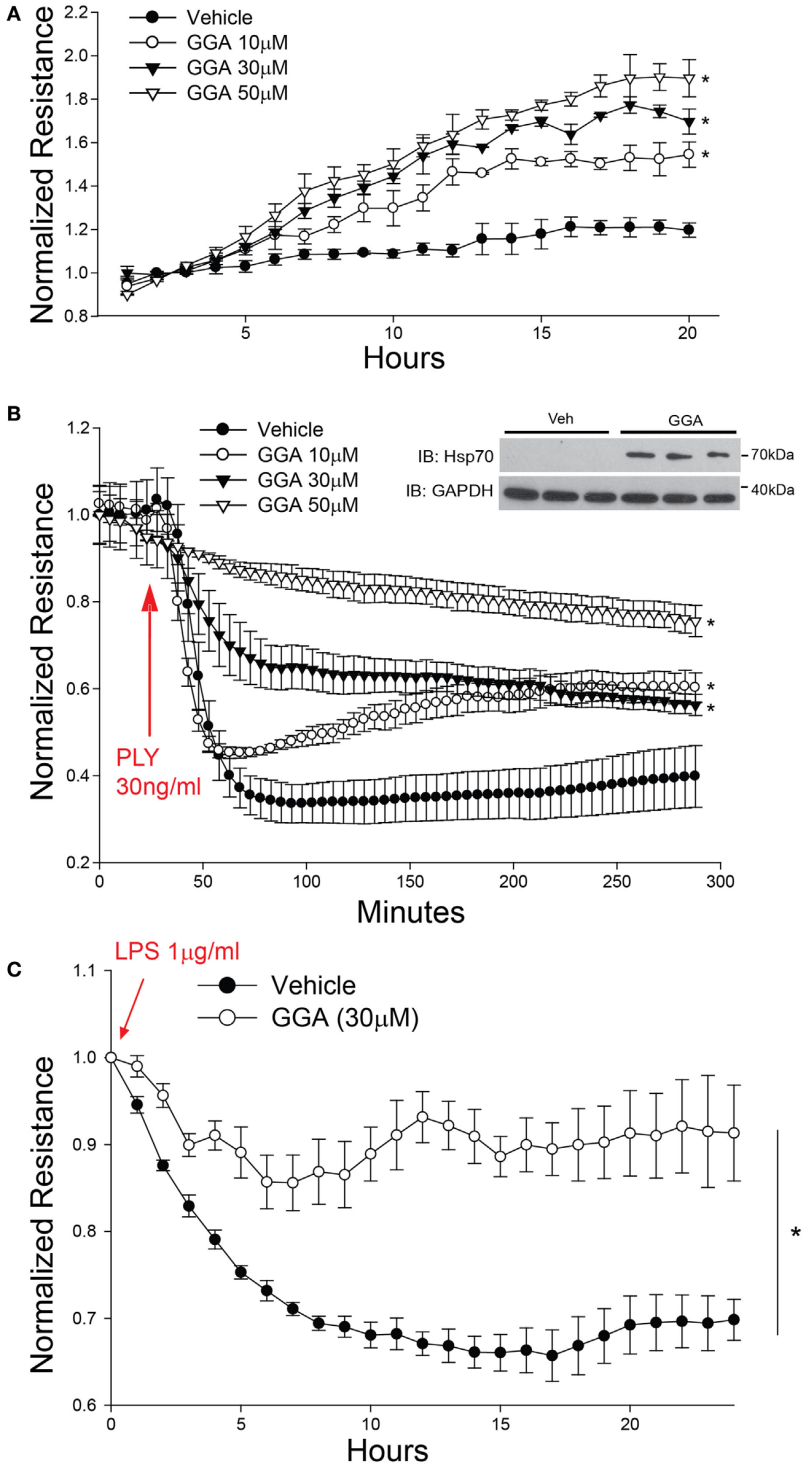
Acute pharmacological upregulation of Hsp70 protects against pneumolysin (PLY) and LPS-induced EC barrier disruption *in vitro*. In panel **(A)** human lung microvascular endothelial cells (HLMVEC) were plated in electric cell-substrate impedance sensing (ECIS) arrays, treated with the indicated concentrations of geranylgeranylacetone (GGA) and changes in barrier strength determined by ECIS over time. Data shown as mean ± SED (*n* = 4 wells for each treatment), *p < 0.05. In panel **(B)** HLMVEC were treated with the indicated concentrations of GGA overnight and then were treated with or without PLY (30 ng/ml) and barrier function determined by ECIS. Data are shown as mean ± SEM (*n* = 4 wells for each treatment). *p < 0.05 versus vehicle. In panel **(C)** HLMVEC were pretreated overnight with GGA (30 µM) and then exposed to LPS (1 µg/ml). Data shown as mean ± SEM (*n* = 4 wells for each treatment). *p < 0.05 versus vehicle (DMSO).

Geranylgeranylacetone (GGA) pharmacologically induces the upregulation of Hsp70 *via* actions on HSF1 to increase *HSP70* gene transcription (43). GGA exhibits low cellular toxicity and has been widely used in Japan for antiulcer therapy (44). HLMVECs treated with GGA overnight exhibited a dose-dependent increase in baseline microvascular barrier electrical resistance (Figure 2A). In contrast, PLY induced an acute and pronounced decrease in HLMVEC resistance, reflecting severe barrier disruption. However, in cells cultured with GGA, there was a dose-dependent protective effect from PLY-induced hyperpermeability (Figure 2B). Gram negative (G^−^) bacterial toxins can also be a significant cause of pneumonia and compromised endothelial barrier function. To assess whether Hsp70 protects against the G^−^ bacterial toxin, LPS, HLMVEC were cultured in ECIS arrays and treated overnight with GGA (30 µM) or vehicle (DMSO). In control cells, exposure to LPS (1 µg/ml) resulted in a more gradual loss of barrier function that peaked at approximately 15 h. In HLMVEC pretreated with GGA, there was a significantly reduced ability of LPS to disrupt endothelial barrier function (Figure 2C).

**Figure 3:**
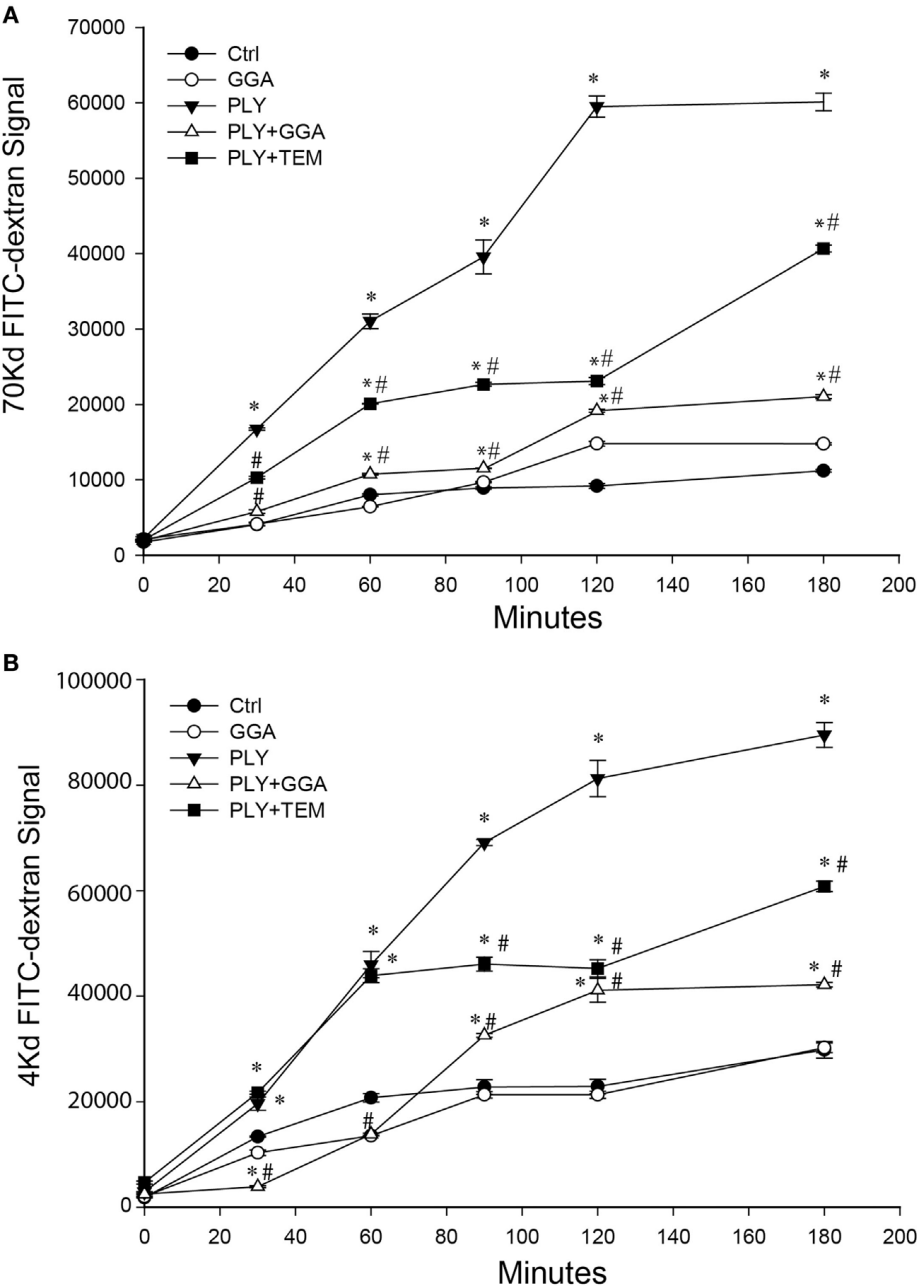
Geranylgeranylacetone (GGA) and TEMPOL protect Human lung microvascular endothelial cell (HLMVEC) from pneumolysin (PLY)-induced barrier disruption. In panel **(A)** 70-kDa FITC-dextran permeability of HLMVEC pretreated with vehicle, GGA (30 µM) or tempol (TEM, 100 μM) then stimulated with PLY (50 ng/ml). *p < 0.05 versus control, ^#^*p* < 0.05 versus PLY. Data shown as mean ± SEM (*n* = 3 wells for each treatment). **(B)** 4-kDa FITC-dextran permeability of HLMVEC pretreated with GGA (30 µM) or tempol (100 μM) then stimulated with PLY (50 ng/ml). *p < 0.05 versus control, ^#^*p* < 0.05 versus PLY. Data shown as mean ± SEM (*n* = 3 wells for each treatment).

PLY-induced EC dysfunction has been associated with its ability to upregulate ROS production (13). To determine the importance of ROS in mediating the loss of barrier function, we employed the superoxide inhibitor, tempol (TEM) in transwell assays. Consistent with previous findings, HLMVECs that were pretreated with GGA for overnight showed a robust decrease in dextran permeability in response to PLY (Figures 3A–B). Although transcellular dextran flux cannot be completely excluded, the decreased passage of both the 4- and 70-kDa FITC-dextran into the lower compartment reflects impaired endothelial monolayer integrity. Inhibition of superoxide with tempol also provided acute protection against PLY-induced barrier disruption (Figures 3A–B). GGA induced upregulation of Hsp70 provided more protection than that afforded by TEM.

### GGA Protects HLMVECs From PLY Caused Mitochondrial Damage

**Figure 4:**
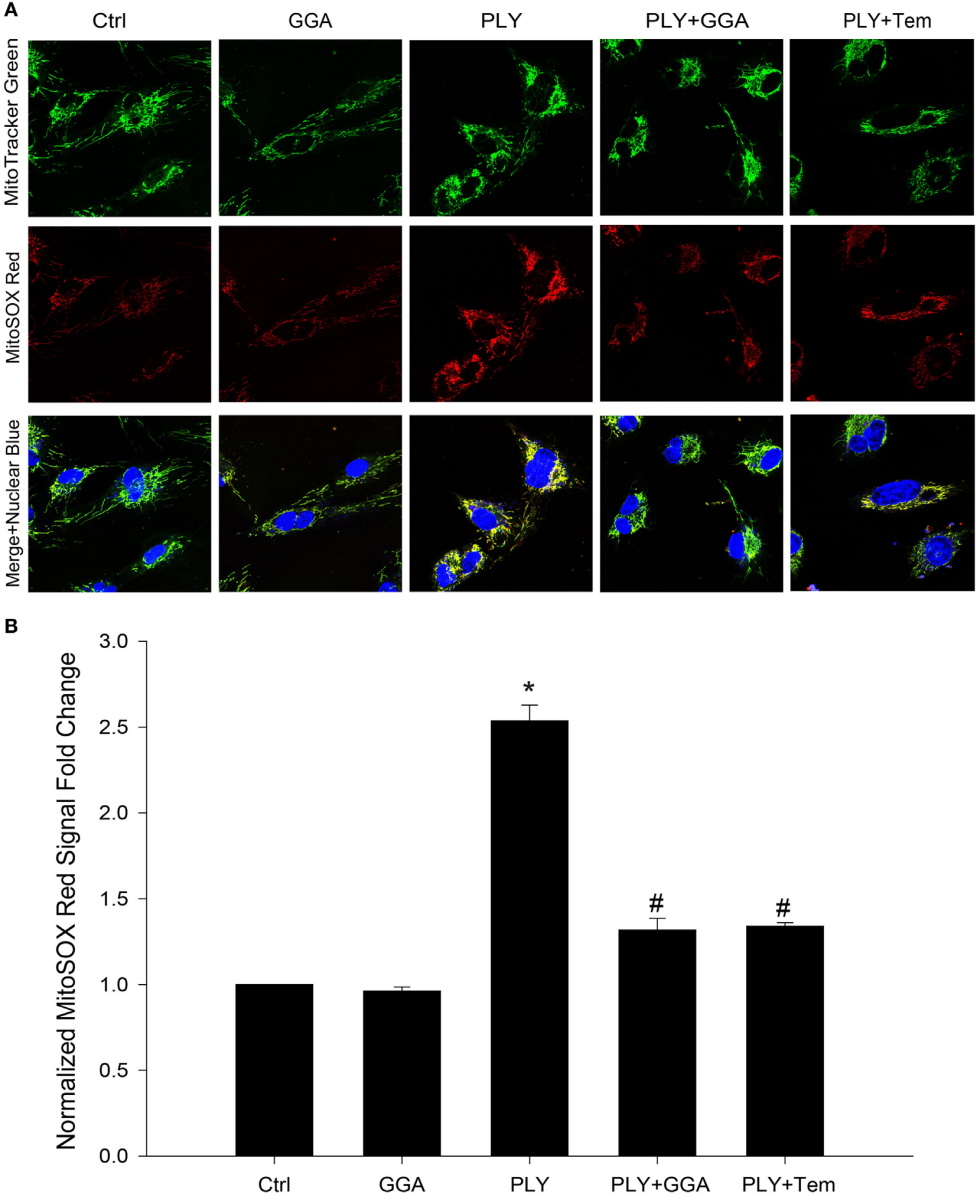
Geranylgeranylacetone (GGA) protects against pneumolysin (PLY)-induced mitochondrial reactive oxygen species (ROS) production. In panel **(A)** representative confocal images of MitoSox red and MitoTracker green stained human lung microvascular endothelial cell reporting the degree of mitochondria localized ROS. In panel **(B)** analysis of fluorescent signal from MitoSox red normalized to MitoTracker green (*n* = 4 wells for each treatment). Data shown as mean ± SEM *p < 0.05 versus control, ^#^*p* < 0.05 versus PLY.

Mitochondria are a major source of ROS production in endothelia cells. Given that Hsp70 and superoxide scavengers can protect HLMVECs from PLY-induced increases in permeability, we next investigated whether the barrier protection afforded by Hsp70 might be mediated through changes in mitochondrial ROS production. HLMVECs were plated on glass bottom dishes, pretreated with 30 µM GGA for 24 h and then loaded with MitoSOX Red and MitoTracker Green. Serum-free medium containing 50 ng/ml PLY or vehicle was added prior to measurement of ROS. Using MitoTracker Green to label mitochondria, we were able to identify that mitochondrial numbers were similar among groups, while red florescence, which represented the ROS produced in the mitochondrial compartment, was significantly increased in HLMVEC stimulated with PLY (Figures 4A,B). In cells that were pretreated with GGA, PLY-failed to stimulate mitochondrial ROS production and cells pretreated with Tempol also demonstrated an attenuation of PLY-stimulated mitochondrial ROS production.

**Figure 5:**
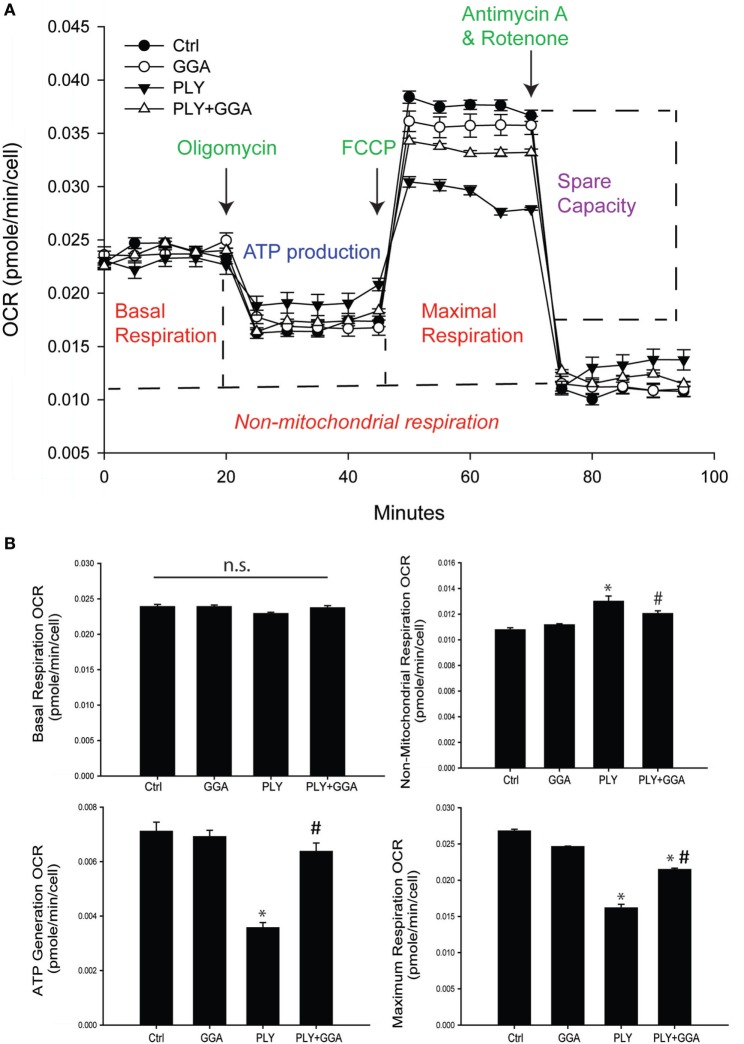
Geranylgeranylacetone (GGA) protects human lung microvascular endothelial cells from pneumolysin (PLY)-induced mitochondrial dysfunction. **(A)** XF96 seahorse Mito stress assay profile with arrows showing the time of injections of oligomycin, carbonyl cyanide *p*-trifluoromethoxy-phenylhydrazone (FCCP), and antimycin A. Data are represented as mean ± SEM (*n* = 5 for each treatment). **(B)** Mitochondria function data were generated using the XF96 seahorse Mito stress assay. Basal respiration OCR (OCR before adding oligomycin-OCR and after adding antimycin A). Non-mitochondrial respiration OCR (stressed OCR after adding antimycin A). ATP generation OCR (basal respiration OCR after adding oligomycin). Maximum respiration OCR (stressed OCR after adding FCCP-stressed OCR after adding antimycin A). Data are shown as mean ± SEM. *p < 0.05 versus control, ^#^*p* < 0.05 versus PLY.

We next assessed whether PLY alters mitochondrial function using the Seahorse extracellular flux analyzer. HLMVECs were pretreated with or without GGA to induce Hsp70 expression and then challenged with or without PLY. Basal rates of mitochondrial respiration were similar among groups (Figures 5A,B). The addition of oligomycin which inhibits ATP synthase (complex V) reduced oxygen consumption in all groups, but less so in cells that were treated with PLY (Figures 5A,B bottom left). This reflects a diminished ability of cells to use oxidative phosphorylation to generate ATP and the preservation of this ability in cells pretreated with GGA. The addition of the protonophore, FCCP [Carbonyl cyanide 4-(trifluoromethoxy) phenylhydrazone] collapses the proton gradient, disrupting the mitochondrial membrane potential, and drives maximal oxygen consumption (complex IV). Maximal respiration rates were decreased in HLMVEC exposed to PLY compared to control and significantly preserved in cells pretreated with GGA (Figure 5B, bottom right). Collectively, these data indicate that PLY compromises mitochondrial respiration and that upregulation of Hsp70 protects against the PLY-induced loss of function.

### GGA Protects HLMVECs From PLY-Induced Cell Death

**Figure 6:**
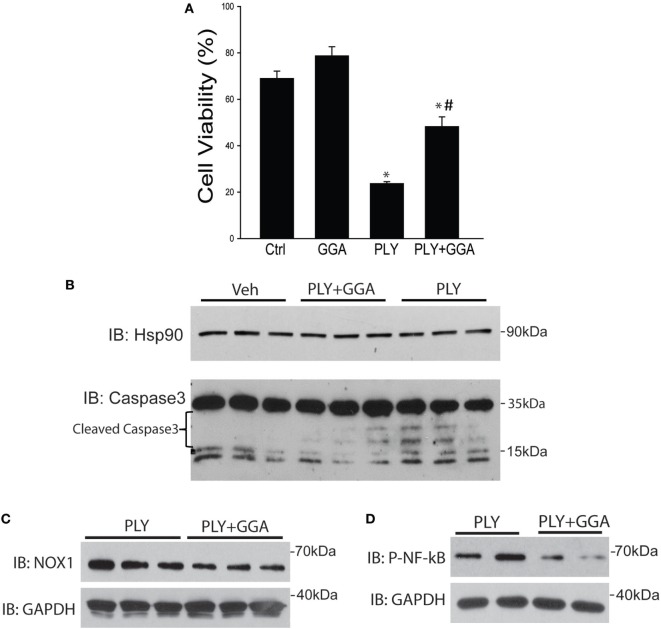
Geranylgeranylacetone (GGA) protects against pneumolysin (PLY)-induced Human lung microvascular endothelial cells (HLMVEC) apoptosis. In panel **(A)** HLMVEC were pretreated 6 h with GGA (30 µM) and then challenged with PLY (50 ng/ml) for 4 h and cell viability determined (MUSE flow cytometry). Data are shown as mean ± SEM, *p < 0.05 versus control, ^#^*p* < 0.05 versus PLY. In panel **(B)** HLMVEC were challenged pretreated with vehicle or GGA (30 µM, 6 h) and then challenged with PLY (50 ng/ml) for 4 h. Cells were lysed and the level of cleaved caspase 3 was determined by western blot (*n* = 3). **(C)** HLMVECs were pretreated with vehicle or GGA overnight and then challenged with PLY (50 ng/ml) for 4 h. Cells were lysed and NOX1 levels determined by western blot. **(D)** HLMVECs were pretreated with vehicle or GGA (30 µM, 6 h) and then challenged with PLY (50 ng/ml) for 4 h. Cells were lysed and levels of phosphorylated (P-) NF-κB p65 were determined by western blot.

Hsp70 is an established cytoprotective factor that can reduce cell death (45, 46). We next assessed whether Hsp70 upregulation decreases cell death in response to PLY. HLMVECs were challenged with PLY and cell viability assessed by Muse flow cytometer. PLY (50 ng/ml) resulted in a significant decrease in cell viability (Figure 6A), which was significantly higher in cells pretreated with GGA (30 µM). To assess whether GGA impacts PLY-induced apoptosis, we evaluated the expression levels of cleaved caspase 3, a commonly used marker of apoptosis in HLMVEC. PLY increased the expression of cleaved caspase 3, an effect that was decreased in cells pretreated with GGA. Neither PLY nor GGA treatment altered the expression levels of total caspase 3 or the loading control, Hsp90 (Figure 6B). Apoptosis can be triggered by both cytosolic ROS and mtROS. MitoSOX assays revealed less mtROS and decreased NOX1 protein expression in HLMVEC treated with GGA (Figure 6C). Given that mitochondrial, and mtROS can both increase inflammatory signaling, we assessed phosphorylated-NF-κB p65 (Ser536) levels in HLMVEC treated with 50 ng/ml PLY with or without GGA. NF-κB p65 phosphorylation was decreased with GGA treatment (Figure 6D).

### GGA Protects Mice From PLY-Induced ALI

**Figure 7:**
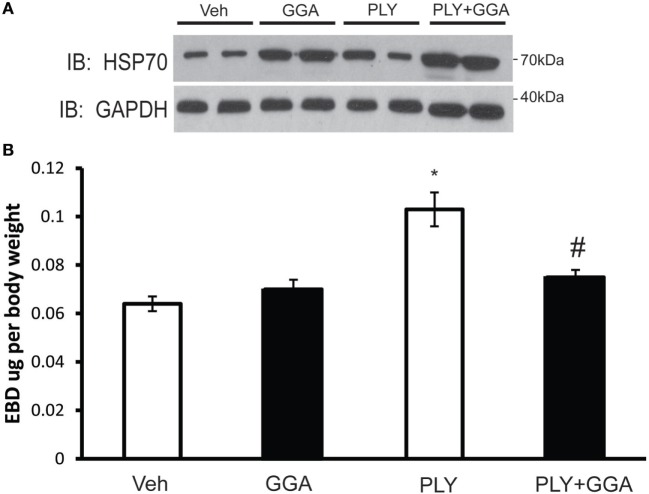
Geranylgeranylacetone (GGA) provides protection against pneumolysin (PLY)-induced vascular leak *in vivo*. Mice were administered vehicle (10% ethanol, IP) or GGA (500 ng/kg, IP) and, 24 h later, were challenged with IT PLY 60 ng/mouse. Evans blue dye-albumin (EBD, 30 mg/kg-2 h) was injected IV *via* the tail vein 2 h before the administration of PLY to assess pulmonary vascular leak (*n* = 5–6). After the mice were sacrificed, lung tissue was lysed and the levels of Hsp70 and GAPDH were determined by Western blot **(A)**, and then the levels of EBD in lung tissue were determined spectrophotometrically at 620–750 nm **(B)**. Data are shown as mean ± SEM, *p < 0.05 versus control, ^#^*p* < 0.05 versus PLY.

To assess the translational relevance of Hsp70 upregulation in a model of ALI, mice were administered GGA (500 mg/kg IP) or vehicle overnight and then challenged with IT PLY (60 ng). Endothelial barrier integrity was assessed by EBD extravasation. A single dose of GGA significantly increased Hsp70 protein expression in lung tissues relative to the loading control, GADPH (Figure 7A). Neither vehicle nor GGA alone influenced baseline levels of EBD in lung tissue, however, in vehicle treated mice, PLY administration evoked a significant increase in pulmonary vascular permeability and extravascular leak of EBD. Pretreatment with GGA significantly blunted the ability of PLY to induce EBD extravasation into the lungs consistent with greater preservation of the pulmonary endothelial barrier (Figure 7B).

## Discussion

Herein, we show that genetic and pharmacological upregulation of Hsp70 provides robust protection from bacterial toxin-induced destruction of the endothelial barrier both *in vitro* and *in vivo*. Increased Hsp70 expression, achieved through adenovirus-mediated gene transfer or activation of HSF with GGA, protected the integrity of the pulmonary microvascular endothelial barrier following challenge with both G^+^ and G^−^ bacterial toxins. The G^+^-toxin PLY promoted increased mitochondrial ROS, decreased mitochondrial function, increased caspase C cleavage, and increased cell death, effects that were mitigated by GGA treatment. In mice, GGA upregulated pulmonary Hsp70 expression and provided significant protection from PLY-induced pulmonary microvascular permeability. Collectively, these results suggest that Hsp70 can protect against bacterial toxin-induced increases in mitochondrial ROS which contribute to the loss of pulmonary endothelial barrier integrity.

Pneumolysin and other cholesterol-dependent pore-forming cytolysins from G^+^-bacteria such as listeriolysin O have been shown to form plasma membrane pores that stimulate calcium entry and promote disruption of the endothelial barrier (12, 13, 41, 42, 47). Multiple mechanisms have been proposed, including activation of PKC, disruption of NO signaling, arginase induction, and inhibition of ENaC. A connecting theme between these mechanisms is the ability of G^+^ toxins to increase ROS, but the sources of superoxide appear to be multifactorial and are incompletely defined (13). PLY has been shown to induce cell death and inflammation in the lung (48) and, in conjunction with elevated ROS it has been shown to promote cell death and apoptosis in multiple cell types including cardiomyocytes (49) epithelial cells (49–51), neurons (15, 52) and cerebral endothelial cells (53), and human umbilical vein endothelial cells (49). The ability of PLY to induce cell death in lung microvascular endothelial cells has not previously been described. Using MitoSOX red, we observed that PLY-stimulated ROS in the mitochondria of HLMVEC. An increase in ROS occurred alongside compromised mitochondrial function and increased cell death. Whether the increase in mitochondrial ROS contributes to PLY-induced cell death is not yet established.

Hsp70 belongs to large family (>13 members) of related 70 kDa Hsp that are both ubiquitous and highly conserved (54). Hsp70 family members share a similar structural organization composed of three basic domains: an N-terminal domain that encodes a highly conserved ATP-binding site, the M or middle domain binds which binds to numerous substrates, and the C-terminal domain facilitates protein folding and the binding of co-chaperones. Some of the Hsp70 genes are constitutively expressed and others are inducible in response to various stressors including increased heat (55), ROS (56), osmolarity (57), and toxins (58) to name a select few. The most abundant Hsp70s are HspA1A (Hsp70-1) and HspA1B (Hsp70-2). To simplify, we will use Hsp70 when referring to HspA1A. Hsp70 is found in the cytoplasm, nucleus, mitochondria, cell membranes, and in the extracellular space (59). Hsp70 influences many aspects of cellular function including binding to nascent proteins, preventing protein aggregation, facilitating protein folding and stability, and regulating protein activity. The upregulation of Hsp70 in response to cell stress provides a survival advantage (46, 60). Hsp70 has been shown to influence cell survival *via* multiple mechanisms (45, 60, 61), which may differ based on the cell type and stimulus. We found that upregulation of Hsp70 provided protection against PLY-induced ROS, mitochondrial dysfunction and ultimately caspase 3 cleavage and cell death. Cell death is an important mechanism underlying ALI (62) and the ability of Hsp70 to provide cell survival may underlie is ability to preserve barrier integrity. While our studies focused primarily on the role of G^+^ toxins, we also showed that Hsp70 upregulation can protect against the loss of barrier function induced by LPS. These data are consistent with other studies in which upregulation of a distinct Hsp70 family member, HspA12B, protects against the loss of barrier function in HUVECs (63). Hsp70 has also been shown to protect against hyperoxia-stimulated loss of endothelial barrier function (39).

Another important finding of our study was the ability of Hsp70 to suppress the production of ROS. Previously, we have shown that upregulation of Hsp70 inhibits the activity of the NADPH oxidases which are the major sources of cellular ROS (35, 64). In this study, we show that Hsp70 suppressed NOX1 expression which is consistent with our previous findings and protects against PLY-stimulated mitochondria ROS. How Hsp70 reduces mitochondrial ROS production in HLMVEC is not yet fully understood and evidence in the literature in other cell types suggest that multiple mechanisms are involved. PLY opens pores in the plasma membrane leading to a pronounced calcium influx that can impair mitochondrial coupling and promote superoxide formation. Within the inner membrane of mitochondria is an ATP sensitive potassium channel (mitoKATP) that can be activated by ATP deprivation and calcium overload. Studies have shown that GGA and Hsp70 reduce mitoROS in cardiomyocytes and protect from ischemia reperfusion injury. The protective effect of increased Hsp70 expression is abolished with the mitoKATP inhibitor, 5-hydroxydecanoate, which does not alter Hsp70 expression. These data suggest that Hsp70 reduces mitoROS accumulation by facilitating the opening of the mitoKATP channel. NADPH derived ROS have been shown to promote oxidative damage of mitochondrial proteins in particularly, complex I and complex II, which results in increased mitoROS production (20). Increased mitoROS can activate NADPH oxidase locking both enzymes into a feed forward relationship (20–22). The upregulation of Hsp70 in HLMVEC would be expected to break this vicious cycle between mtROS and NADPH oxidase through actions on both pathways. Hsp70 has also been shown to attenuate oxidative phosphorylation (65) which may reduce mitoROS generation. Hsp70 can also impair mitochondrial proteostasis (66) which may compromise local antioxidant (SOD2) pathways or improve the function of enzymes in the electron transport chain. Hsp70 has also been shown to inhibit NADPH oxidase activity (35) which can secondarily impact mitoROS. Finally, increased expression of Hsp70 has been shown to upregulate Akt-eNOS activity and the resulting increase in NO could indirectly suppress mitochondrial ROS by quenching superoxide (67).

Others have shown that Hsp70 can protect against cell death by mechanisms upstream of the mitochondria and by suppressing the ability of ROS (H_2_O_2_) to induce mitochondrial dysfunction (68, 69). There is little Hsp70 (1A1) in the mitochondria of normal cells; however, tumor cells have significantly increased amounts which provide a survival advantage. Depletion of Hsp70 in tumor cells disrupts mitochondria function and increases cell death (70). There is also a constitutive mitochondrial isoform of Hsp70 (HspA9 or motalin) which has important roles in protein translocation and although it is not induced by stress, it has also been shown to be important for cell survival (71). In our study, we upregulated Hsp70 using GGA and using adenoviral mediated gene transfer of a specific Hsp70 (HspA1A). How Hsp70 traffics to the mitochondria is not completely understood. Hsp70 can interact with lipids and in particular cardiolipin which is localized to the mitochondria (72). In addition to actions on Nox enzymes and the mitochondria, Hsp70 upregulation is also associated with reduced inflammation and the upregulation of antioxidant pathways (73). Inhibition of mitochondrial complexes I, II, and III has been shown to protect cardiomyocytes from hypoxia induced injury by reducing p38 phosphorylation as well as inflammatory signaling (74). Interestingly, blockade of ROS diffusion in the mitochondria using anion inhibitors also protects cardiomyocytes (74), which suggests an important role of mtROS in the initiation of inflammation during conditions of cellular stress. As the pulmonary inflammatory response can be very different in young versus elderly patients (28), future studies may want to compare the protective effects of Hsp70 in both young and aged animal models of PLY-induced ALI. Previously, we and others have shown that increased intracellular calcium is important in mediating the loss of endothelial barrier function in response to PLY (13, 75). Whether Hsp70 upregulation alters calcium-dynamics in HLMVEC is not yet known. Recent studies have revealed that PLY can activate TLR4 (76, 77) which is also the primary target of LPS. However, the kinetics of the loss of barrier function induced by PLY and LPS are quite different suggesting different mechanisms and furthermore, Hsp70 has been shown to both support and stimulate TLR4 signaling (78).

Hsp90 inhibitors have been shown to protect the endothelial barrier from G^−^ bacterial toxins and are potent anti-inflammatory agents (79, 80). However, Hsp90 inhibitors also upregulate Hsp70 and the prosurvival actions of Hsp70 limit the effectiveness of these agents in anticancer strategies (81). In acute settings, whether the effectiveness of Hsp90 inhibitors relates to their ability to upregulate Hsp70 remains to be determined. Hsp70 has been shown to be released into the extracellular space through a yet to be identified mechanism. Increased circulating Hsp70 levels reflect heightened inflammation and poor outcome and autoantibodies against Hsp70 have been observed in a number of diseases (59). Although, upregulation of Hsp70 may promote cell survival and potentially increase extracellular Hsp70, the negative effects of long-term upregulation appear to be minor. GGA is approved for use as an antiulcer medication in Japan and appears to have low toxicity. Any potential risks are expected to be lower in the short-term treatment of ALI.

In conclusion, our study reveals that Hsp70 upregulation is a rapid and potent modality to protect the pulmonary endothelial barrier from the G^+^ bacterial toxin, PLY. Attractive features of this approach include the ability to rapidly upregulate Hsp70 with pharmacological agents and a broad spectrum of protection against both G^+^ toxins, G^−^ toxins and hyperoxia. Hsp70 also targets multiple pathways including mitochondrial function, ROS production, and cell death which are key mechanisms that underlie the loss of endothelial barrier integrity. Upregulation of Hsp70 may be of high clinical significance in the management of ARDS/ALI-related pulmonary barrier dysfunction.

## Ethics Statement

All animal studies conformed to National Institutes of Health guidelines. The experimental procedure was approved by the Augusta University Institutional Animal Care and Use Committee.

## Author Contributions

XL: conceptual ideas, performed experiments, and wrote paper. YY and BG: performed experiments. SH and ZB: critical reading and performed experiments. DW, RR, SB, AV, and YS: critical reading. TC: provided reagents. RL: conceptual ideas, reagents, and critical reading. DS and DF: conceptual ideas and critical reading. FC: conceptual ideas, experiments, and critical reading.

## Conflict of Interest Statement

The authors declare that the research was conducted in the absence of any commercial or financial relationships that could be construed as a potential conflict of interest.
